# Editorial: Omics in respiratory virus infectious diseases: integrating multi-omics to reveal data characteristics and mechanisms for the diagnosis and treatment of disease

**DOI:** 10.3389/fmed.2023.1273662

**Published:** 2023-09-20

**Authors:** Xuerui Wang, Luis Ulloa

**Affiliations:** ^1^Beijing Hospital of Traditional Chinese Medicine, Capital Medical University, Beijing, China; ^2^Beijing Key Laboratory of Basic Research with Traditional Chinese Medicine on Infectious Diseases, Beijing, China; ^3^Beijing Institute of Chinese Medicine, Beijing, China; ^4^Department of Anesthesiology, Duke University Medical Center, Durham, NC, United States

**Keywords:** multi-omics, respiratory infection, virus infection, pneumonia - clinical features and management, respiratory viral infections

Viral infection accounts for 90% of acute respiratory infections producing symptoms from pharyngitis to bronchitis and pneumonia. Viral pneumonia is caused by the infection and invasion of the upper respiratory tract by viruses such as the influenza virus, parainfluenza virus, syncytial virus, cytomegalovirus, adenovirus, rhinovirus, coronavirus, and some enteroviruses. These viruses can cause major outbreaks, such as the epidemic caused by COVID-19, which killed nearly seven million people worldwide in 3 and half years. Severe pneumonia is often accompanied by multiple organ failure and high mortality sepsis. In this special issue, we collected several critical studies highlighting the applications of multi-omics technology in respiratory viral infections. Multi-Omics include a wide diversity of novel techniques from genomics, proteomics, metabolomics, transcriptomics, lipidomics, immunohistochemistry and glycomics that are providing critical insights about the pathogenesis of these infections and novel therapeutic targets.

A study conducted by Tongyoo et al. recruited 2,153 patients and reported the clinical outcomes, complications, and predictive factors of hospitalized patients diagnosed with respiratory syncytial virus (RSV) infection using a significant cohort of the same period. Among the patients with respiratory failure, the 28-day mortality, ventilator-dependent days, and hospital-admission days were similar for patients with and without RSV infection. However, patients with RSV infection had an increased risk of complications, such as bronchospasm, ventilation-associated pneumonia (VAP), and lung atelectasis. Acute respiratory distress syndrome (ARDS), VAP, and prolonged ventilator days were independently associated with subsequent in-hospital death, whereas antiviral therapy was not associated with decreased mortality. Tracheostomy was identified as a factor associated with reduced in-hospital mortality. This study provides strong evidence to monitor respiratory complications including VAP and lung atelectasis in these patients, particularly in those with mechanical ventilation. The study also implies that a tracheostomy should be performed as early as possible when prolonged airway or mechanical ventilation support is needed.

In the study reported by Spurbeck et al., they compared metatranscriptomic sequencing of Illumina, MinION, and hybridization-based sequencing methods to determine the most effective high throughput sequencing method for pathogen tracking in wastewater. Wastewater-based epidemiology has been used for decades to track viral spread throughout communities, especially during the COVID-19 pandemic. This study has great economic and epidemiological significance for the expansion of the wastewater monitoring network that was used during the COVID-19 period to monitor other future infectious diseases. They recommend targeted hybridization, which was able to detect bacterial, fungal, and viral pathogens in the wastewater, as a viable singular method for known pathogen surveillance. This study also provides critical information about the standardization and optimization process of wastewater monitoring, including positive controls for each targeted pathogen, expanded hybridization panel of all reportable infectious diseases, determination of the optimal workflow to detect all pathogens, and determination of the detection limit for each pathogen in wastewater.

The critical implications of awake prone position (APP) have become even more relevant during the COVID-19 pandemic. Many recent RCTs, systematic reviews and meta-analyses have confirmed its critical implications and the absence of indisputable scientific evidence in favor or against APP. A meta-analysis with 14 RCTs of 3,290 patients reported by Peng et al. concluded that the APP was beneficial to improve the oxygenation of patients with ARDS or acute hypoxic respiratory failure (AHRF) caused by COVID-19 and to reduce the need for intubation. The study reported no evident survival advantage, hospital length of stay, ICU admission, and adverse events as compared with the usual care. The main results of this meta-analysis are consistent with recent publications by Weatherald et al. (1). However, Peng et al. found that APP significantly improved the SpO_2_/FiO_2_ ratio in patients with COVID-19. This difference may be due to the different patient populations, mainly including a report on an analysis of 501 patients (2), as it did not meet strict RCT standards and was not included in this study. In summary, this meta-analysis shows strong evidence supporting the beneficial use of APP in COVID-19 patients.

Given the complex natural characteristics of traditional Chinese medicine (TCM) herbs, their multi-target and multi-pathway regulatory effects and mechanisms have been become more relevant clinically. In many cases, the efficacy of the treatment is limited by the absorption of the active ingredients and their spread into the bloodstream. Many components act through the secondary digestion of gut microbiota, and partially through metabolites of the microbiota. Therefore, gut microbiota is considered as the second organ for digestion, absorption, and efficacy of the treatment. Multi-omics that combine gut microbiota and metabolomics are very useful for exploring the multi-target and depicting the overall mechanisms of TCM to improve efficacy. Lian et al. first identified 18 major active compounds in plasma after oral administration of the Qin-Qiao-Xiao-Du (QQXD) formula. The effects of QQXD formula were shown with improved survival, weight loss, lung viral load, lung injury, and cytokines relative in influenza-infected mice. They reported improved gut microbiota dysbiosis increased intestinal carbohydrate metabolism and up-regulated cyanoamino acid metabolism pathway. This complete study reveals the therapeutic effects and mechanism of the new Chinese herbal compound QQXD on influenza and its association with the microbiota and metabolites.

In general, the studies of this special issue provide critical insights for the application of multi-omics technology in respiratory infectious diseases. We look forward to the growth of this exciting and clinically relevant research of omics techniques to reveal the mechanisms of respiratory viral infections, identify susceptible populations, and their applications for epidemiologic and environmental monitoring.

## Author contributions

XW: Writing—original draft. LU: Writing—review and editing.
